# *ALK*-rearranged pulmonary adenocarcinoma in Thai Patients: From diagnosis to treatment efficacy

**DOI:** 10.1186/s12957-016-0893-6

**Published:** 2016-05-03

**Authors:** Pimpin Incharoen, Thanyanan Reungwetwattana, Sakditad Saowapa, Kaettipong Kamprerasart, Duangjai Pangpunyakulchai, Lalida Arsa, Artit Jinawath

**Affiliations:** Department of Pathology, Faculty of Medicine Ramathibodi Hospital, Mahidol University, Bangkok, Thailand; Division of Medical Oncology, Department of Medicine, Faculty of Medicine Ramathibodi Hospital, Mahidol University, Bangkok, Thailand; Faculty of Medicine Ramathibodi Hospital, Mahidol University, Bangkok, Thailand

**Keywords:** pulmonary adenocarcinoma, *ALK* gene rearrangement, Crizotinib, efficacy

## Abstract

**Background:**

Anaplastic lymphoma kinase (*ALK*) gene rearrangement is detected in 3 % to 13 % of non-small cell lung carcinoma patients, and these patients benefit from ALK inhibitors. The aim of this study was to determine the prevalence, the clinical and histological characteristics and the treatment outcomes of ALK-rearranged lung adenocarcinoma using immunohistochemistry (IHC) IHC, reverse transcription polymerase chain reaction (RT-PCR) and fluorescence in situ hybridization (FISH) methodologies.

**Methods:**

A total of 268 pulmonary adenocarcinoma patients were screened for ALK expression by ALK IHC, which was confirmed by FISH and/or RT-PCR for ALK gene rearrangement. The treatment outcomes of ALK-rearranged patients were retrospectively reviewed.

**Results:**

*ALK* gene rearrangement was identified in 26 cases (9.7 %) with no EGFR co-mutation, and it showed significant associations with younger age, female sex and non-smoker status (p < 0.05). A cribriform growth pattern was identified as the dominant histologic feature, and a solid signet ring cell component was focally present in a minority of the cases. Among 12 ALK-rearranged patients with conventional treatment, seven cases in the early stage of disease were cured and alive, and five patients in the late stage of the disease progressed and died, with a median overall survival (OS) at 14 months. Of the 14 patients receiving crizotinib, all of them had clinical benefit from crizotinib treatment, with one patient having a complete response (CR), 12 patients having a partial response (PR) and one patient having stable disease (SD). On the cutoff date, six of 14 patients were continuing crizotinib treatment with a median time of response of 7.5 (3–13) months, while eight patients had disease progression, and five of them died with a median OS at 8 months.

**Conclusion:**

*ALK* gene rearrangement tended to occur in younger, non-smoking, female patients. ALK IHC is a reliable screening method to detect *ALK* gene rearrangement. Crizotinib therapy provided treatment benefit in ALK-rearranged adenocarcinoma patients especially in advanced stages of the disease.

## Background

Lung cancer is a leading cause of cancer-related death worldwide [1, 2]. Non-small cell lung cancer (NSCLC), particularly adenocarcinoma, is the most common subtype of lung cancer diagnosed today [3]. The standard treatment is surgical resection in early stages and palliative chemotherapy/radiation in the advanced stages. Over the past few years, somatic mutation of epidermal growth factor receptor (*EGFR*) has been detected in 10-76 % of NSCLC cases, particularly among East Asian female non-smoking patients, leading to EGFR tyrosine kinase inhibitor-targeted therapy [4–6]. Moreover, novel fusion genes, including anaplastic lymphoma kinase (*ALK*) and echinoderm microtubule-associated protein-like 4 (*EML4*) on chromosome 2 or *ALK* with other partner genes, have been observed [7–10]. This *ALK* gene rearrangement has been identified in 3-13 % of NSCLC patients and is significantly associated with young, female, non-smoking patients and with adenocarcinoma subtype with solid signet ring cell features and advanced stage of disease [11–40]. Moreover, *ALK* gene rearrangements are mutually exclusive from *EGFR* gene mutations in NSCLC. Crizotinib was the first approved ALK inhibitor. Ceritinib and alectinib are second-generation ALK inhibitors that are already approved by the US FDA and Japan FDA, respectively [41, 42].

Despite a wide variety of translocation patterns, fluorescence in situ hybridization (FISH) can detect *ALK* gene rearrangement regardless of translocation form. This technique has been used in several clinical ALK inhibitor trials and has become a gold standard for *ALK* rearrangement detection. However, FISH is limited because it has an expensive probe set, and it requires an optimal fixation method with a complicated assay. Immunohistochemistry (IHC) and reverse transcription polymerase chain reaction (RT-PCR) have been used as alternative methods for *ALK* gene rearrangement detection [30, 43]. IHC can generally be performed in most routine laboratories. It is easy to interpret and inexpensive. Different IHC clones and scoring criteria for ALK expression in NSCLC have been studied by several authors. Antibody clones 5A4 (Novocastra, Newcastle Upon Tyne, UK) and D5F3 (Ventana, Tuczon, AZ, USA), with additional detection systems, appear to be more sensitive and more specific than the ALK1 (Dako) clone. Recently, the US FDA approved an IHC companion diagnostic developed by the Roche subsidiary Ventana Medical Systems (ALK (D5F3) CDx Assay) [44]. The RT-PCR method has been performed successfully with considerable sensitivity and specificity in fresh frozen cancer specimens, but it has had limited performance with formalin-fixed paraffin-embedded (FFPE) tissue. This technique is the best method for detecting *ALK* rearrangement in cytology specimens, such as bronchial lavage, brushing or washing, or body cavity fluid. However, the RTPCR method also requires multiple primer sets to cover all possible fusion variants. Several studies have reported well-correlated results among IHC, FISH and RT-PCR in detecting *ALK* gene rearrangement, while other studies have demonstrated complex discrepancies [23–40].

In the present study, the authors applied IHC, FISH and RT-PCR to detect *ALK* gene rearrangement. A total of 268 lung adenocarcinoma patients were screened for ALK expression by IHC using the anti-ALK antibody D5F3 clone. Then, the IHC-positive cases were confirmed by FISH and RT-PCR for *ALK* gene rearrangement. The prevalence of ALK-rearranged pulmonary adenocarcinoma in Thai patients; the correlations among the IHC, FISH and RT-PCR techniques; and the treatment outcomes of this patient group are discussed.

## Methods

### Patients and samples

The study considered 268 cases of lung adenocarcinoma diagnosed between January 2009 and December 2014. The FFPE tissue blocks were selected from the archives of the Department of Pathology of Ramathibodi Hospital. Age, sex, smoking history and disease stage for all cases were obtained from the medical records. Tumor histology and treatment outcomes were reviewed in the ALK-rearranged cases.

The present study was approved by the Committee on Human Rights Related to Research Involving Human Subjects, Faculty of Medicine, Ramathibodi Hospital, Mahidol University.

### IHC

Sections of FFPE tissue measuring 4 mm in thickness were stained with the mouse monoclonal antibody for ALK using the prediluted Ventana anti-ALK rabbit monoclonal antibody (clone D5F3), together with the Optiview DAB IHC detection kit and an Optiview amplification kit on a Ventana Benchmark XT stainer (Ventana Medical Systems, Tucson, AZ, USA).

Immunoreactivity was evaluated as positive when the tumor showed diffuse or multifocal granular cytoplasmic staining with strong intensity.

### RT-PCR

The RNA was extracted from FFPE tissues using a High Pure FFPE RNA Isolation Kit (Roche Diagnostics, Mannheim, Germany). *EML4-ALK* fusion transcripts were detected using the AmoyDx EML4-ALK fusion gene detection kit (Amoy Diagnostics, Xiamen, China). Fusion variants 1, 2, 3a, 3b, 4, 4′, 5a, 5b, 5′, and 8 were detected in 3 reactions according to the manufacturer’s protocol.

### FISH

The *ALK* FISH was performed on unstained, 4-μm FFPE tissue sections. The *ALK* break-apart FISH was performed using the Vysis *ALK* Break Apart FISH Probe kit (Abbott Molecular Inc., Abbott Park, IL, USA). The LSI ALK 5' probe (Spectrum Green) and the LSI ALK 3' probe (Spectrum Orange) were used. The hybridization and assessment were performed with standard controls. At least 50 tumor cells without equivocal or single signals were scored. Cells with separated signal in ≥ 2 signal diameters or cells with isolated 3' (red) signals were defined as *ALK* rearrangement-positive cells. The tumor was defined as *ALK* gene rearrangement positive when the rearrangement-positive cell rate was ≥ 15 % of the cells. EGFR mutational status was also recorded when available.

### Treatment outcomes and efficacy

The outcomes and treatment efficacy were retrospectively reviewed in the ALK-rearranged patients. Patients treated with crizotinib received a drug dose of 200–250 mg twice daily. Tumor evaluation was performed by reviewing the CT scan using the Response Evaluation Criteria in Solid Tumors (RECIST), version 1.1 [45], at baseline and every 8 weeks thereafter. The overall best response was reported as complete response (CR), partial response (PR), stable disease (SD) or progressive disease (PD). The cutoff date of the study was June 1, 2015.

### Statistical analysis

The association of *ALK* rearrangement status with clinicopathological data was analyzed by Pearson's Chi-square test, Fisher's exact test as appropriate and Wilcoxon’s rank-sum test for continuous data. All of the statistical analyses were performed using SPSS software, version 18.0 (SPSS, Chicago, IL, USA), and significance was set at p < 0.05.

## Results

### Clinicopathological characteristics of ALK-positive patients

A total of 268 cases included 139 surgically resected and 129 biopsied specimens. One hundred fifty-one (56.3 %) cases were of female patients, and 117 (43.7 %) cases were of male patients. The median age was 63 years old, ranging from 24 to 90. The pathologic stages were I, II, III and IV in 80 (29.8 %), 40 (14.9 %), 42 (15.7 %) and 106 (39.6 %) patients, respectively.

Among the 268 patients, 26 (9.7 %) cases were ALK-positive after being screened by IHC and confirmed by FISH and RTPCR. The clinicopathological characteristics between *ALK*-positive and *ALK*-negative adenocarcinoma cases are summarized in Table 1. ALK gene rearrangement was significantly more common in younger, female, non-smoking patients (p = 0.006, 0.036 and 0.032, respectively). Disease stage had no significant differences (p = 0.158) between the two groups.

**Table 1:** Clinicopathological comparison between *ALK* gene rearrangement-positive and -negative pulmonary adenocarcinoma

Variable	Patient total (*n* = 268)	ALK rearrangement		*P* value
		Positive (*n* = 26)	Negative (*n* = 242)	
Age				0.006
Median	63	59.5	63	
Range	24–90	24–90	24–89	
Gender				0.036
Male	117 (43.7)	6 (23.1)	111 (45.8)	
Female	151 (56.3)	20 (76.9)	131 (54.2)	
Smoking				0.032
Never smoking	202 (75.4)	24 (92.3)	177 (73.1)	
Smoking	66 (24.6)	2 (7.7)	65 (26.9)	
Stage				0.540
Early (I–II)	120 (44.6)	10 (38.5)	110 (55)	
Advanced (III–IV)	148 (45.4)	16 (61.5)	132 (54.5)	

**Fig. 1:**
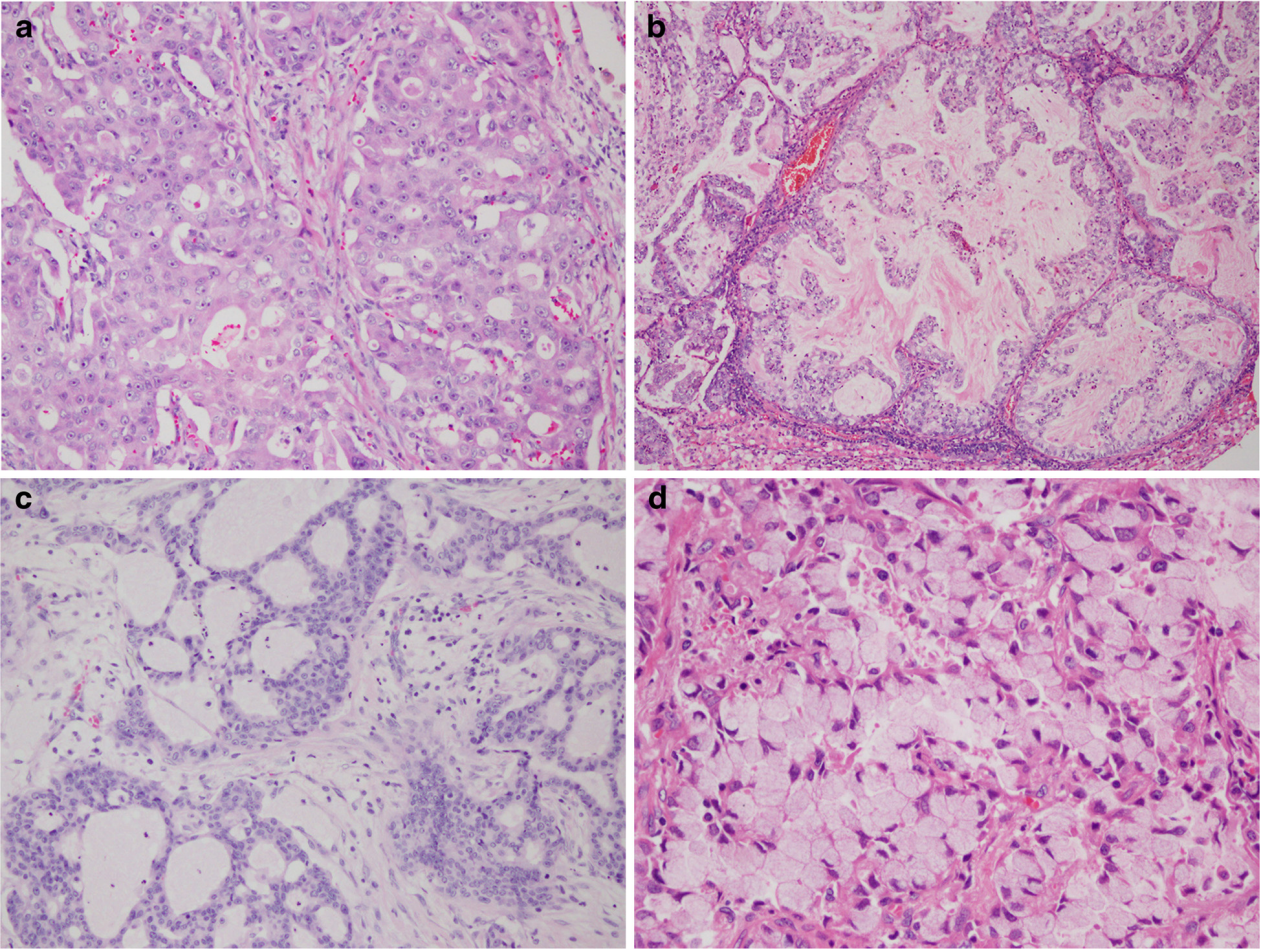
Histologic features of ALK-rearranged cases. Most of the cases demonstrated a cribriform growth pattern (**a**) with abundant extracellular mucin production (**b**, **c**). Few cases contained focal solid signet ring cell features (**d**)

Tumor histology was reviewed in 11 surgically resected cases of the 26 ALK-positive cases. Among these 11 cases, the predominant growth pattern was a cribriform pattern in 4 cases, an acinar pattern in 2 cases, a solid pattern in 2 cases, and a papillary and micropapillary pattern in 1 case each; one case showed invasive mucinous adenocarcinoma. A cribriform growth pattern was a predominant feature in most of the cases and was also identified focally in all of the cases. Solid signet ring cell features were identified as a minor component in 2 cases (Fig 1). None of the cases had a predominantly lepidic growth pattern.

### *ALK* gene rearrangement detection by IHC, FISH and RT-PCR

**Fig. 2:**
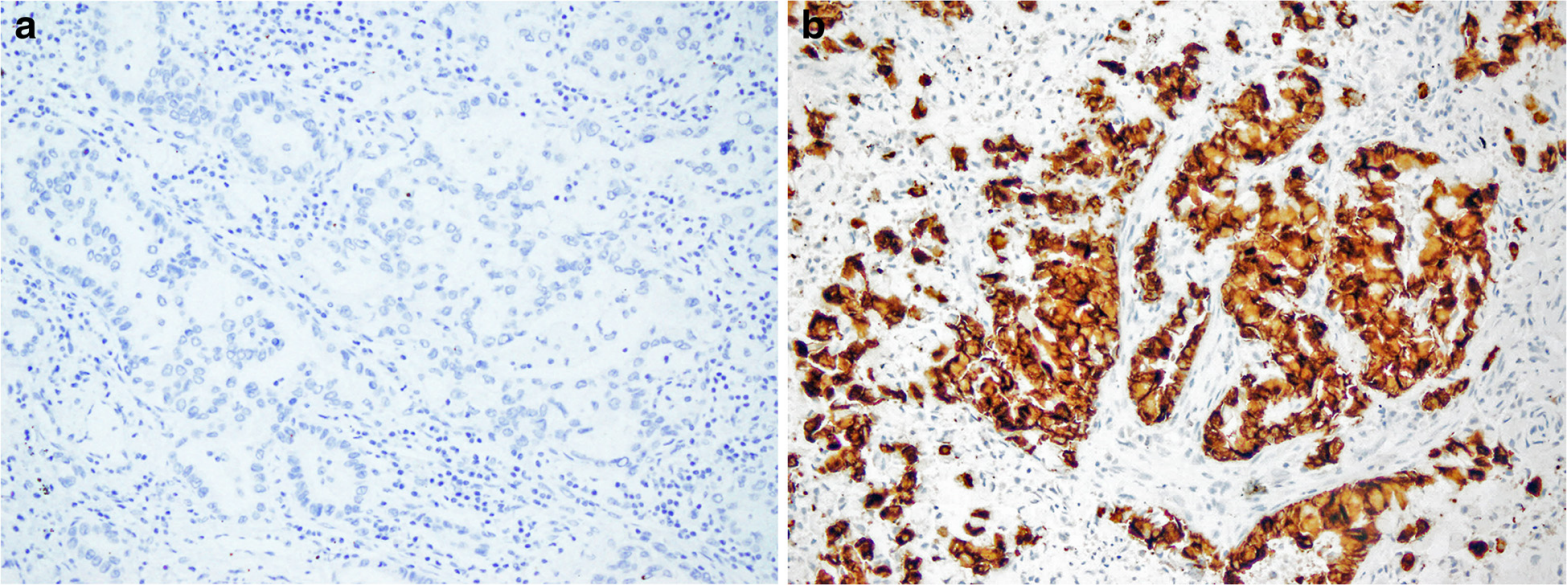
Examples of the IHC results. An ALK IHC-negative case (**a**) and an ALK IHC-positive case demonstrating diffuse, strong membranous and cytoplasmic staining (**b**)

**Fig. 3:**
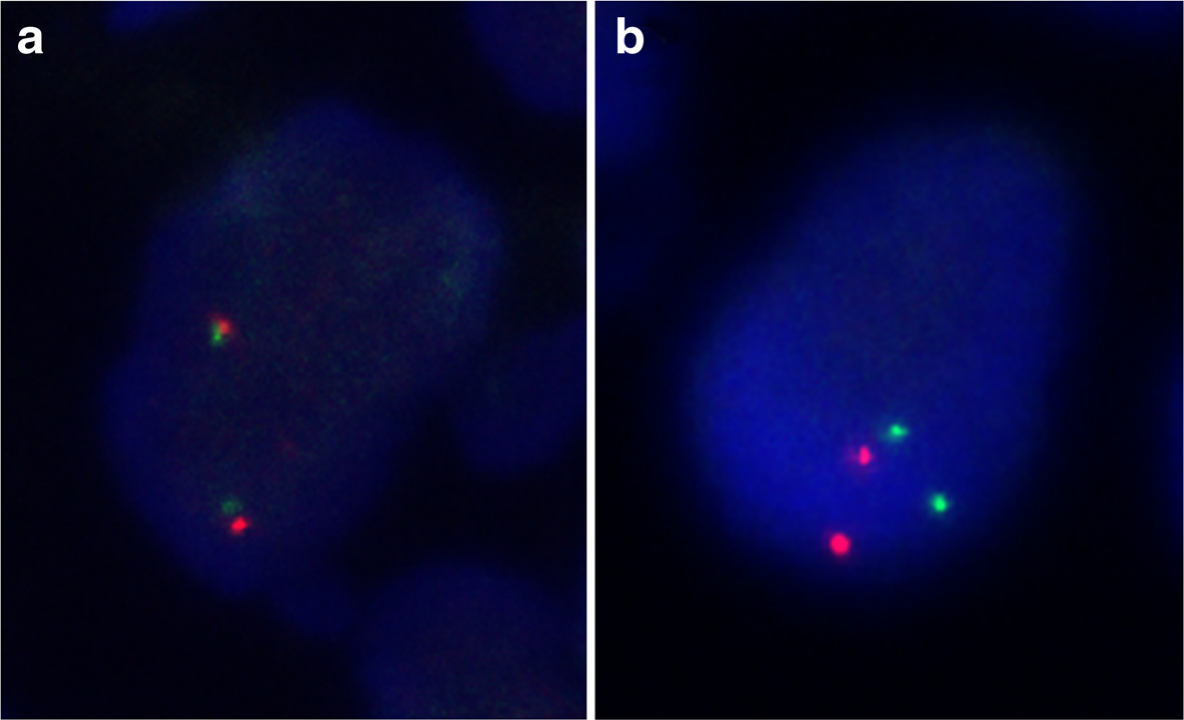
Examples of the FISH results. Tumor cells negative for ALK rearrangement demonstrated fused green and red probes (**a**), and tumor cells positive for ALK rearrangement demonstrated separate green and red probes (**b**)

The results of IHC, FISH and RT-PCR in 26 cases are demonstrated in Table 2. Among the 26 ALK IHC-positive cases, one case contained insufficient tumor cells for FISH, 4 cases failed FISH due to no hybridization signal, and 20 cases showed *ALK* gene rearrangement positivity by FISH. One case was negative by FISH due to the percentage of break-apart cells that did not meet the criteria for diagnosis (Figs. 2 and 3). FISH results were available in 21 ALK IHC-negative cases, and all of them were negative for ALK gene rearrangement. The correlation between IHC and FISH was evaluated in these 42 cases and is demonstrated in Table 3. The sensitivity and specificity of IHC compared with FISH were 100 % and 95 %, respectively. The positive and negative predictive values for IHC were 95 % and 100 %, respectively.

**Table 2:** Clinicopathologic characteristics and treatment outcomes of ALK-rearranged patients

Case	Age	Sex	Stage	SS	IHC	FISH	RT-PCR	Treatment	Outcome	Status
1	60	F	I	NS	+	Fail	Fail	Surgical resection + adjuvant CMT	CR	Alive
2	69	F	I	NS	+	Fail	+	Surgical resection	CR	Alive
3	56	F	I	NS	+	Fail	+	Surgical resection + adjuvant CMT	CR	Alive
4	41	F	I	NS	+	+	+	Surgical resection	CR	Alive
5	69	F	I	NS	+	+	+	Surgical resection	CR	Alive
6	43	F	II	NS	+	+	+	Surgical resection + adjuvant CMT	CR	Alive
7	47	F	II	NS	+	+	+	Surgical resection + adjuvant CMT	CR	Alive
8	62	F	IV	NS	+	+	+	Lost to follow-up	NA	Alive
9	45	M	IV	NS	+	−	+	Best palliative care	PD	Death
10	51	M	IV	NS	+	+	+	CMT + WBRT	PR	Death
11	43	F	IV	NS	+	Fail	−	Surgical resection + CMT + WBRT	PR	Death
12	40	F	IV	NS	+	+	+	Surgical resection + RT	PR	Death
13	63	F	I	NS	+	+	+	Surgical resection + CMT + third-line crizotinib	PR	Alive
14	71	F	I	NS	+	+	+	Surgical resection + CMT + fourth-line crizotinib then ceritinib	PR	Alive
15	63	F	II	NS	+	+	+	Surgical resection + CMT + second-line crizotinib	PR	Alive
16	49	M	III	NS	+	+	+	First-line crizotinib	PR	Alive
17	61	F	III	NS	+	+	+	CMT + third-line crizotinib	CR	Alive
18	69	M	IV	NS	+	+	+	CMT then third-line crizotinib	PR	Alive
19	27	F	IV	NS	+	+	+	First-line crizotinib then ceritinib	PR	Alive
20	61	F	IV	NS	+	+	+	CMT + second-line crizotinib	PR	Alive
21	74	M	IV	SM	+	+	+	CMT + second-line crizotinib	PR	Alive
22	61	F	IV	NS	+	+	−	CMT + second-line crizotinib	SD	Death
23	90	M	IV	SM	+	+	+	First-line crizotinib	PR	Death
24	40	F	IV	NS	+	+	+	CMT + third-line crizotinib	PR	Death
25	38	F	IV	NS	+	+	+	CMT + third-line crizotinib	PR	Death
26	24	F	IV	NS	+	NA	+	First-line crizotinib	PR	Death

*Abbreviations: SS* smoking status, *NS* non-smoker, *SM* smoker, *CR* complete response, *PR* partial response, *SD* stable disease, *PD* progressive disease, *NA* not available

**Table 3:** Correlation between ALK IHC and FISH on the available 42 cases

IHC	FISH	
	Positive	Negative
Positive	20	1
Negative	0	21

In the 26 ALK IHC-positive cases, 25 cases had adequate RNA quality for further RT-PCR analysis by assessment of the internal control *PGK* transcripts, and an *EML4-ALK* fusion transcript was identified in 23 of 25 cases.

EGFR mutational data were available in 184 cases; 73 (39.7 %) cases were EGFR mutation positive, and 111 cases had the wild-type EGFR. Among the 26 ALK-positive cases, EGFR mutational status was available in 22 cases, and none of these cases contained EGFR mutations. The incidence of *ALK* gene rearrangement increased up to 20 % in wild-type EGFR patients (22/111 cases).

### Treatment outcome and crizotinib efficacy

Of the 26 ALK-positive patients, one patient was lost to follow-up; 11 patients (42 %) received surgical resection or surgical resection plus chemotherapy, chemotherapy alone or the best palliative care, depending on the stage of the disease and performance status without crizotinib therapy. Fourteen patients (54 %) received crizotinib therapy at various time points: four patients (28.5 %) as first-line treatment, four patients (28.5 %) as second-line treatment, five patients (36 %) as third-line treatment, and the other one (7 %) as fourth-line therapy (Table 4). Two patients received ceritinib after systemic failure of crizotinib.

**Table 4:** Median time of response in *ALK*-rearranged adenocarcinoma patients treated with crizotinib

Treatment line	Time of response, month^a^, range (median)
First-line (*N* = 4)	2–16 (5.5)
Second-line (*N* = 4)	3–13 (5.5)
Third- and fourth-line (*N* = 6)	1–10 (8)

^a^Cutoff date was 1 June 2015

Seven (64 %) of 11 patients receiving conventional treatment (not receiving crizotinib) in the early stage of the disease were in CR and still alive, with a median time from diagnosis to the study cutoff date of 54 months, while four (36 %) patients in the advanced stage had PD or died, with a median OS of 21 months. All 14 of the patients receiving crizotinib had clinical benefits from crizotinib treatment, and one patient had CR, 12 patients had PR, and one was a SD patient. The median times of response in the patients receiving crizotinib as their first-line, second-line and late-line (third and fourth) treatments were 5.5, 5.5 and 8 months, respectively, without significant differences (p = 0.84) (Table 4). At the cutoff date, six of 14 patients were continuing with crizotinib treatment: one was a first-line treatment patient, three were second-line treatment patients, and two patients were third-line treatment patients with a median time of response of 7.5 (3–13) months. Two patients developed progressive disease and were taking ceritinib at the time this report was written. One patient who developed disease progression in the brain was bedridden and being treated with the best palliative care only at the cutoff date. Five patients with multiple distant metastases had disease progression and died after 1 week to 6 months of crizotinib treatment, with a median OS of 8 months. Interestingly, one patient developed an aggressive flare of brain metastases 12 days after stopping crizotinib.

## Discussion

*ALK* rearrangement is the one of most frequent molecular alterations in NSCLC, particularly adenocarcinoma. In previous studies, *ALK* rearrangement was detected in 3-13 % of NSCLC patients, and this occurrence was associated with younger age, non-smoking, advanced stage disease and the presence of signet ring cell morphology [11–40]. In this study, the prevalence of ALK-rearranged adenocarcinoma patients was similar to previous published data and was significantly associated with younger patients, females and non-smoking status. Although more than half of the ALK-positive patients were in stage IV of the disease (15/26), no significant association between disease staging and *ALK*-rearranged status was identified in our study. *ALK* gene rearrangement did not coexist with EGFR gene mutation in our study. Although prior studies have reported some cases with concurrent ALK and EGFR or KRAS alterations [15, 16, 25, 37, 46, 47], this event might occur in a minority of cases; additionally, this study had the limitation of unavailable EGFR and KRAS mutational status. In terms of histology, cribriform pattern was the most frequent predominant growth pattern, rather than the solid signet ring cell pattern, which was focally found in only two cases. Although these two distinct morphologies were characteristic of ALK-rearranged NSCLC in prior studies [16, 18–22], the cribriform growth pattern appears to be more frequent in Asian populations than the solid signet-ring cell pattern, which is more common in Western populations [19, 20]. Moreover, in this study, ALK-rearranged adenocarcinoma showed no lepidic growth pattern, which was prevalent in several prior studies [16, 18–22].

FISH is now a standard and acceptable method to detect ALK rearrangement in pulmonary adenocarcinoma. However, the cost per test is very high, and the test requires a special technique and interpretation. In many studies, the correlation between IHC and FISH methods was calculated to apply IHC as a screening method for ALK rearrangement detection.

Some ALK IHC clones are commercially available for use. Both ALK IHC clones 5A4 (Novocastra, UK) and D5F3 (Ventana, USA) have shown high sensitivity and specificity, ranging from 83 % to 100 % [23–40]. In this study, the researchers performed D5F3 clone with Optiview and an Optiview amplification kit and found 100 % sensitivity and 95 % specificity, in agreement with previous studies using the same clone and detection system [17, 35, 36]. The ALK IHC in our study was very easy to interpret without variations in stain intensity or background disturbances in most cases, so we deferred to the interpretation criteria from the manufacturer's protocol.

Discordance between FISH and IHC in our study was found in one case, which was IHC and RT-PCR positive while FISH was negative. False-negative FISH results have been documented in prior studies. One study showed a case with less than 15 % break-apart tumor cells similar to the false negative case in our study (14 % break-apart cells) [28]. Another study demonstrated complex gene rearrangement by next generation sequencing, which is unable to detect rearrangements that are observable by break-apart FISH [48]. The RT-PCR assay used in the present study consisted of most common fusion variants that covered 90 % of *EML4-ALK* rearranged cases. Therefore, the two RT-PCR negative cases in our study might represent rare minor *EML4-ALK* fusion variants or other partners. However, RT-PCR demonstrated *ALK* rearrangement in one case that provided insufficient tumor cells for FISH and in two cases that showed uninterpretable signals by FISH. In addition to each technical limitation, the quality of DNA and RNA in the FFPE tissue blocks also affected the analysis outcomes. The FFPE blocks used in this study were collected from 2009 to 2014, and all of the tests were performed in 2014. Four ALK-positive cases diagnosed between 2009 and 2010 were unamplified by FISH, and RNA could not be extracted from one block for RT-PCR.

In terms of disease outcomes, *ALK*-rearranged adenocarcinoma patients who were in the early stages and had resectable disease showed excellent prognosis even without crizotinib therapy. Moreover, patients in the advanced stages of the disease, especially stage IV with multiple distant organ metastases, showed poorer outcomes regardless of the therapeutic method used. Therefore, the outcome of treatment depended on the stage of the disease, rather than ALK status.

Regarding the efficacy of crizotinib, our study showed a comparable median time of response to that in previous studies [49–55]. Furthermore, crizotinib is still efficient as a late line of treatment in advanced disease. In Thailand, reimbursement is allowed for crizotinib patients who are in second- or late-line treatment and patients who have the Civil Servants Reimbursement plan only; otherwise, the patients must pay out of pocket. The flare phenomenon was also found in *ALK* positive patients who received crizotinib and then stopped the drug due to the progressive nature of the disease, as discussed in previous reports of EGFR TKI treatment [56, 57]; therefore, the flare phenomenon should be noted in all patients who fail ALK inhibitor treatment.

## Conclusions

Screening IHC, followed by FISH or RT-PCR in IHC-positive cases, was found to be the most cost-effective method. Having several available methods in the laboratory would be very helpful for identifying or confirming *ALK* gene rearrangement in equivocal cases or cases with sample limitations. Crizotinib therapy possesses clinical benefits for *ALK*-rearranged adenocarcinoma patients, comparable with previous studies.
